# Polycaprolactone Fiber and Laminin and Collagen IV Protein Incorporation in Implants Enhances Wound Healing in a Novel Mouse Skin Splint Model

**DOI:** 10.1155/2024/2515383

**Published:** 2024-09-04

**Authors:** Dina Gadalla, Maeve Kennedy, Jamie Ganem, Mustafa Suppah, Alessandra Schmitt, David G. Lott

**Affiliations:** ^1^ Head and Neck Regenerative Medicine Laboratory Mayo Clinic Arizona, Phoenix 85054, AZ, USA; ^2^ Department of Laboratory Medicine & Pathology Division of Anatomic Pathology Mayo Clinic Arizona, Phoenix 85054, USA; ^3^ Department of Otolaryngology Division of Laryngology Mayo Clinic Arizona, Phoenix 85054, USA

## Abstract

Wound healing is an intricate process involving multiple cells and distinct phases, presenting challenges for comprehensive investigations. Currently available treatments for wounds have limited capacity to fully restore tissue and often require significant investments of time in the form of repetitive dressing changes and/or reapplications. This article presents a novel study that aims to enhance wound healing by developing biomaterial scaffolds using Medpor®, a porous polyethylene implant, as a model scaffold. The study incorporates electrospun poly(e-caprolactone) (PCL) fibers and a protein mixture (PM) containing collagen IV and laminin onto the Medpor® scaffolds. To evaluate the impact of these implants on wound healing, a unique splinted wound model in mice is employed. The wounds were evaluated for closure, inflammation, collagen deposition, angiogenesis, epithelialization, and proliferation. The results show that wounds treated with Medpor® + PCL + PM implants demonstrate accelerated closure rates, improved epithelialization, and enhanced angiogenesis compared to other implant groups. However, there were no significant differences observed in collagen deposition and inflammatory response among the implant groups. This study provides valuable insights into the potential benefits of incorporating PCL fibers and a PM onto scaffolds to enhance wound healing. Furthermore, the developed splinted wound model with integrated implants offers a promising platform for future studies on implant efficacy and the advancement of innovative wound healing strategies.

## 1. Introduction

Wound healing is a complex process involving various cells and phases, which make it challenging to study. Regardless of the cause of wounds, be it trauma, surgery, or disease, they cause damage to the local tissue and its surrounding environment [1]. The healing of the tissue is mediated by extracellular matrix (ECM) remodeling, soluble mediators, and dynamic cellular pathways [2]. Typically, wound healing is categorized into three stages: inflammation, proliferation, and remodeling [3, 4]. Disruptions during the wound healing process, such as interleukin deficiency, diabetes, or senescence, can lead to acute or chronic tissue abnormalities, and current treatment options often require multiple surgical interventions, dressing changes multiple times a day, and are limited in their ability to restore tissues, posing a burden on wound healing [5]. Therefore, there is a need for novel methodologies to enhance wound healing and improve patient outcomes, making tissue regeneration a significant area of interest in regenerative medicine [6].

There are several regenerative medicine approaches to wound healing, including growth factors, stem cells, and biomaterials [7, 8]. While growth factors are easy to apply, they are subject to rapid degradation, and while stem cells are easy to isolate and have self-renewal capacity, they are not effective in large wound areas [9]. Biomaterial scaffolds incorporate bioactive factors in three-dimensional environments that mimic the native ECM, holding the most promise for wound healing strategies [10, 11]. Medpor®, a porous, high-density polyethylene implant, has been safely used as a scaffold for tissue reconstruction in various anatomical regions [12, 13]. However due to its inherent lack of bioactivity, further research and improvements are necessary to enhance the regenerative capacity of Medpor® implants. To evaluate potential enhancements to traditional scaffolds, this study utilizes Medpor® as an experimental scaffold, which has a porous structure allowing tissue and blood vessel ingrowth, as well as nonantigenic properties that minimize the risk of infection or rejection. Furthermore, incorporating additional biological materials, such as proteins, extracellular vesicles, and growth factors, to functionalize the surface of Medpor® implants may further enhance physiological repair mechanisms [13–15].

Electrospinning is a promising technique to enhance scaffold design by generating continuous, fibrous meshes whose structure more closely mimics the native ECM [16]. Poly(e-caprolactone) (PCL), a nontoxic, nonreactive, and cost-effective polyester biomaterial, has gained attention in tissue engineering due to its ability to be fine-tuned to individual patient needs [17]. However, PCL alone is bioinert, necessitating the incorporation of bioactive elements for skin regeneration [18]. PCL meshes, which are typically porous and degrade slowly, have been investigated for biological substance release in wound healing models that require extended periods of time for healing [3, 4, 19, 20]. For optimal tissue growth, bioactive components should mimic the ECM, specifically the attachment site for epithelial and endothelial cells, known as the basement membrane [21]. Collagen IV and laminin, key components of the ECM, have shown promise for wound healing due to their presence in basement membranes and their roles in cell migration, adhesion, and proliferation [21–23]. Utilizing Medpor® as a model scaffold, and combining it with electrospun PCL with a protein mixture (PM) containing collagen IV and laminin, the feasibility of a clinically relevant biomaterial combination for *in vivo* implantation can be explored. The design of such a multifaceted scaffold aimed to couple the beneficial mechanical characteristics of Medpor® with the advantageous morphological properties of electrospun PCL and the important bioactive properties of the PM.

To accurately investigate wound healing mechanisms and evaluate the effect of implants *in vivo*, preclinical animal models are required. Mouse models are a common tool in aiding the scientific community's understanding of wound healing mechanisms, yet it is crucial to consider the structural differences between human and rodent skin. Rodents have an additional subcutaneous striated muscle layer called the panniculus carnosus, which rapidly contracts the skin following wounding [24]. In contrast, human wound healing primarily relies on re-epithelialization and granulation tissue formation for closure, as opposed to contraction [25]. To overcome this difference and better mimic human wound healing, splints can be utilized in rodent models to prevent local skin contraction and instead promote healing by re-epithelialization and granulation tissue formation. Although the splinted model has been used for investigating open-wound closure, there are very little literature studies using this model with an implanted material placed in the wound opening. Given the growing popularity of biomaterial implants, it is crucial to explore adapting the splinted model to an implant setting.

This study aims to evaluate the effect of incorporating PCL fibers with or without ECM proteins onto Medpor® scaffolds. In addition, it seeks to enhance an existing splinted wound model that can accurately evaluate the effect of implants on wound healing. The mechanisms involved in wound healing, including epithelialization, granulation tissue formation, and angiogenesis, will be assessed and quantified. This model is expected to provide valuable insights for future studies investigating implants for wound healing and other therapeutic modalities.

## 2. Materials and Methods

### 2.1. Chemicals and Reagents

Secondary antibodies, 4,6′-diamidino-2-phenylindole (DAPI), and ProLong™ glass antifade mountant were obtained from Thermo Fisher Scientific (Waltham, MA). Polycaprolactone (PCL) (Mn 80 kDa) and collagen IV from human placenta were obtained from Sigma-Aldrich (Saint Louis, MO). Extra pure 2,2,2-trifluoroethanol (TFE) was obtained from Thermo Fisher Scientific (Suwanee, GA). Human recombinant laminin 332, heparin sodium from porcine intestinal mucosa, and saponin were obtained from BioLamina (Sundbyberg, Sweden), Meitheal Pharmaceuticals (Chicago, IL), and EMD Millipore (Billerica, MA), respectively. High-density porous polyethylene Medpor® sheets of 0.25 mm thickness (catalog# 83020) were purchased from Stryker Corp. (Kalamazoo, MI). Primary antibodies against Ki67 (ab16667), CD31 (ab56299), and wide spectrum cytokeratin (ab9377) were obtained from Abcam Inc. (Cambridge, MA), while antibodies against E-cadherin (E-CAD, 610181) and alpha smooth muscle actin (*α*-SMA, NBP1-30894) were obtained from BD Biosciences (Franklin Lakes, NJ) and Novus Biologicals (Centennial, CO), respectively.

### 2.2. Animals

All animal studies were conducted under Mayo Clinic Institutional Animal Care and Use Committee (IACUC) approval. Twelve 8-week-old C57BL/6 mice were obtained from Jackson Laboratory (Bar Harbor, ME). There were four mice per experimental group, comprised of two males and two females. Mice were housed in a temperature- and light-controlled animal facility within the Mayo Clinic Department of Comparative Medicine with free access to sterile food, water, and bedding. Mice were housed in groups upon arrival but were housed individually after the procedure to ensure that postoperative dressings remained intact.

### 2.3. Implants

  Medpor®: Circular punches measuring 6 mm in diameter were cut from Medpor® sheets to use as implants. These implants are henceforth denoted as Medpor®.
  Medpor® + PCL: To prepare the electrospun fiber layers, a 10 wt% polymer solution composed of a 99%/1% (w/w) mixture of PCL and heparin in a TFE solvent was used. The PCL and heparin solutions were prepared and combined as previously described [26]. The mixture was vigorously stirred overnight at room temperature. Subsequently, the solution was loaded into a 10 mL plastic syringe and connected to a programmable syringe pump as part of a Spraybase set up (Spraybase, Cambridge, MA). Electrospinning was carried out with a feed rate of 3 mL/h using a 20-gauge stainless steel needle emitter. A high-voltage generator was used to charge the solution to 15 kV. PCL fibers were collected for 15 min on Medpor® implants that were secured to a grounded rotating collector (1300 rpm) placed 15 cm away from the needle tip to produce a fibrous mesh layer over the implants [27]. Then, these implants, referred to as Medpor® + PCL henceforth, underwent a sterilization process. They were left to air-dry overnight at room temperature, and then soaked in ethanol (70% v/v) overnight. Subsequently, they were incubated in distilled water overnight, exposed to UV light for 30 min on the fiber side, and finally immersed in PBS overnight.
  Medpor® + PCL + PM: A protein mixture (PM) comprising laminin and collagen IV was adsorbed onto the surface of Medpor® + PCL implants. The implants were immersed in a PM solution containing laminin (10 *μ*g/mL) and collagen IV (20 *µ*g/mL) in PBS at 37°C overnight. Following incubation, the implants were thoroughly washed twice with sterile PBS to remove any unbound protein. These modified implants are designated as Medpor® + PCL + PM for future reference.

### 2.4. In Vivo Wound Study

**Figure 1:**
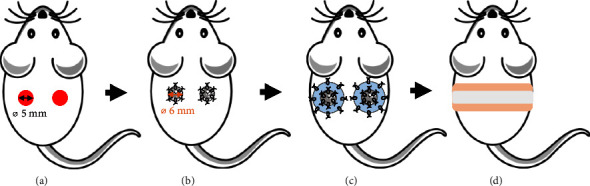
Full thickness excisional wound model illustrating (a) wound creation, (b) implant placement, (c) silicone splint placement, and (d) bandage and tape wrapping.

Light anesthesia was induced using 3% isoflurane with 1 L/min oxygen flow, for just enough time to subcutaneously administer a single dose (3.25 mg/kg body weight) of buprenorphine extended release to all animals. After at least thirty minutes, anesthesia was reinduced, and the dorsum of each mouse was shaved with an electric clipper. A depilatory agent was then applied for 30 seconds and then wiped off to completely remove all hair. The mice were transferred to a sterile field, and surgeries were performed using aseptic techniques. Anesthesia was maintained using 1.5% isoflurane with 1 L/min oxygen flow delivered via face mask. The surgical field was sterilized using three rounds of betadine swabs followed by alcohol wipes. A drape was applied to expose only the surgical field. All instruments were sterilized via autoclave prior to the procedures. Using biopsy punches, two identical 5 mm circular wounds were created bilaterally on each side of the mouse's midline, just below the scapula (Figure 1(a)). The wound sizes were intentionally made slightly smaller than the implants, as wound sizes tend to become slightly larger than their initial sizes. Microscissors were used to ensure the removal of the epidermis, dermis, hypodermis, and panniculus carnosus. Then, 6 mm diameter implants, either Medpor®, Medpor® + PCL, or Medpor® + PCL + PM, were placed in the wounds and sutured to the surrounding skin using six 8-0 Ethilon-interrupted sutures (Figure 1(b)), taking care to minimize unnecessary handling of the implants. The fiber side of Medpor® + PCL and Medpor® + PCL + PM implants faced the exterior environment. Donut-shaped silicone splints were applied to the wound edges using cyanoacrylate adhesive and reinforced with eight 6-0 prolene-interrupted sutures (Figure 1(c)). To protect the implants and keep the wound from drying out, a clear semiocclusive adhesive dressing (3M Tegaderm) was cut into 2 × 2 cm squares. In the center of each square, a thicker donut-shaped silicone ring was placed. This construct was positioned with the thick silicone ring facing the wound, creating a tenting effect on the dressing and preventing it from touching the implant surface. A 1-inch-wide piece of vet wrap bandage was wrapped around the mouse's abdomen and secured with a surgical cloth tape (Figure 1(d)) to prevent the implants from being disturbed by chewing or scratching. At the completion of surgery, the animals were placed on a heating pad and closely monitored until they fully recovered from anesthesia.

#### 2.4.1. Postoperative Care

Animals were monitored daily for 21 days for general ambulatory ability, signs of distress, and behavioral changes. They were weighed every other day at the time wound photos were taken. At the end of the 21-day study, all animals were euthanized via carbon dioxide inhalation. The wounds were excised with an additional margin of normal skin around the wound edges and fixed in 10% formalin overnight.

### 2.5. Wound Analysis

Wound characteristics were assessed grossly throughout the study and evaluated histologically after surgery. All analyses were conducted in a blinded manner to avoid any bias.

#### 2.5.1. Wound Photographs and Area Measurements

Digital images of the wounds were taken starting from day 0 (the day of surgery) and then every other day, with the Tegaderm and vet wrap dressings being changed each time. Images were obtained using a Dino-Lite Edge Digital Microscope (AnMo Electrics Corporation), and DinoCapture 2.0 software was used to assess the healed areas of the wounds over the study period. Wound areas were measured by tracing the circumference of the wound using a wireless Bamboo Touchpad (Wacom, Kazo, Japan) in ImageJ (National Institutes of Health). The wound areas were calculated as a percentage of the original wound area using the following formula:

(1) $$\begin{array}{c} \text{Wound area at day}\times \left(\%\text{ of original size}\right)=\frac{{\text{wound area}}_{\text{Day }x}}{{\text{wound area}}_{\text{Day }0}}\times 100. \end{array}$$

Complete wound closure, represented by a 0% wound area value, was defined as the point at which the implant was completely covered with new tissues. The choice of representative wounds within each group for figures was made while remaining blinded to both the experimental groups being tested and the objectives of this study.

#### 2.5.2. Histological Analysis

Histological analyses were conducted to evaluate the wounds. All harvested wounds were embedded in paraffin. Serial sections were cut into 5 *µ*m thickness and mounted onto slides for histological analysis. Sections obtained from the center of the implant/wound were examined. Hematoxylin and Eosin (H&E) staining, Picrosirius red staining, CD31 and *α*-SMA immunofluorescent double staining, Ki67 and E-CAD immunofluorescent double staining, and cytokeratin immunofluorescent staining were performed to analyze the pathological characteristics of the implants.

H&E slides were imaged using light microscopy, and the inflammatory response was evaluated. The analysis was conducted by a pathologist who was blinded to the experimental group of each sample. A scoring system measuring inflammation was employed, with scores ranging from 0 to 4 [28]. A score of 0 indicated no inflammation, while a score of 1 indicated that 0–25% of the wound was infiltrated by inflammatory cells. Scores of 2, 3, and 4 indicated that 25–50%, 50–75%, and more than 75% of the wound area, respectively, was infiltrated. Picrosirius red-stained wounds were imaged under polarized light to visualize the deposited dermal collagen. The collagen content was quantified by measuring the amount of Picrosirius red-stained tissues under polarized light and expressed as a percentage of the total wound area. The alignment of collagen fibers was determined by measuring the angles of 800 fiber segments relative to the wound bed surface orientation over eight image fields/samples. Angles were then converted to alignment values ranging 0-1 (0 for nonaligned 90° angle fibers and 1 for parallel aligned 0° angle fibers).

For immunofluorescently stained slides, paraffin sections on slides were dewaxed, and antigen retrieval was performed with a citrate-based buffer under pressure for 15 min at 110°C. Slides were then blocked with protein block solution (DAKO) and 0.1% saponin for 30 min. Primary antibodies were then added in antibody diluents with a background reducing component (DAKO) for 1 h at room temperature. The dilutions for CD31, cytokeratin, *α*-SMA, E-CAD, and Ki67 antibodies were 1 : 100, 1 : 300, 1 : 200, 1 : 200, and 1 : 500, respectively. Slides were rinsed three times with PBS before adding secondary antibodies. For E-CAD and Ki67 stainings, a mixture of 1 : 200 goat anti-rabbit IgG highly cross-adsorbed conjugated with Alexa fluor 488 (A11008) and 1 : 500 goat anti-mouse IgG highly cross-adsorbed conjugated with Alexa Fluor 546 (A11030) secondary antibodies was used. For other dual stainings, a mixture of 1 : 200 goat anti-rabbit IgG highly cross-adsorbed conjugated with Alexa fluor 488 and 1 : 200 goat anti-rat IgG highly cross-adsorbed conjugated with Alexa Fluor 546 (A11081) secondary antibodies was used. Antibodies were diluted in the antibody diluent and kept on slides for 30 min at room temperature. Subsequently, slides were washed twice with PBS and exposed to DAPI for 10 min to facilitate cell nuclei counterstaining. Slides were then rinsed again with PBS, coverslipped using ProLong™ glass antifade mountant, and placed in a dark chamber to dry overnight at 4°C. Images were taken with an Olympus IX83 Microscope System. Using ImageJ, the percentage of positive CD31, *α*-SMA, and Ki67 cells was determined from immunofluorescence staining by counting the number of fluorescent-stained cells and the total number of DAPI-positive nuclei in the same wound area.

### 2.6. Statistical Analysis

Numeric variables were assessed for the normality of their distribution using the Shapiro–Wilk test of normality. Normally distributed data values are presented as mean ± standard deviation (SD). Statistical analysis was performed using ANOVA with Tukey's HSD post hoc test in StatPlus LE software (AnalystSoft, Walnut, CA). Non-normally distributed data are presented as median (interquartile range (IQR)) and statistically analyzed using the Mann–Whitney *U* tests. A significance level of *p* < 0.05 was used to determine statistical significance and was marked as follows: ^∗^*p* < 0.05 and ^∗∗^*p* < 0.01.

## 3. Results

All animals experienced no surgical complications, and throughout the study duration, all mice maintained a normal weight and did not display any signs of distress.

### 3.1. Wound Closure

**Figure 2:**
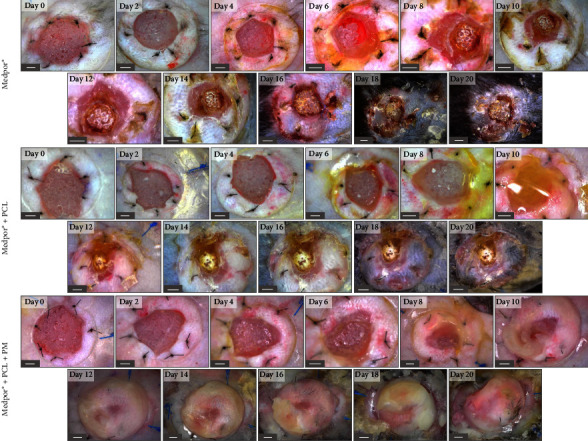
Comparison of wounds at days 0, 2, 4, 6, 8, 10, 12, 14, 16, 18, and 20 in C57BL/6 mice receiving Medpor®, Medpor® + PCL, and Medpor® + PCL + PM implants. Scale bars correspond to 1 mm.

**Figure 3:**
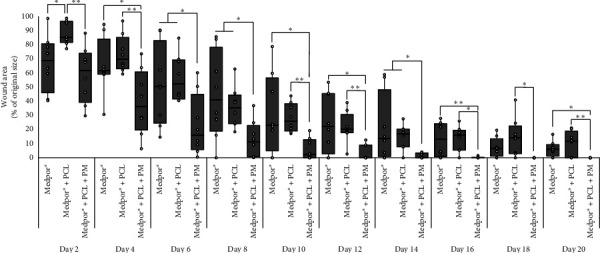
Kinetics of wound healing 2–20 days postwounding in C57BL/6 mice receiving Medpor®, Medpor® + PCL, and Medpor® + PCL + PM implants. Boxes present median and IQR. Mann–Whitney *U* tests were used in statistical analysis for *n* = 8 (number of wounds), ^∗^*p* < 0.05 and ^∗∗^*p* < 0.01.

To assess the impact of implants on wound healing, we utilized three types of implants: (a) Medpor®, (b) Medpor® + PCL, and (c) Medpor® + PCL + PM. Wound photographs were captured every other day for each of the implant groups (Figure 2), and wound sizes were quantified (Figure 3). This allowed us to observe the progressive closure of the wounds. Wounds receiving Medpor® + PCL + PM implants demonstrated faster closure compared to wounds receiving Medpor® and Medpor® + PCL implants (Figure 3), as evidenced by their complete closure between days 12 and 14. In contrast, wounds receiving Medpor® and Medpor® + PCL implants had areas remaining uncovered by tissues (Figures 2 and 3), even by day 20.

On day 4, the wound area in the Medpor® + PCL + PM group (36.1 (19.6–60.7)%) was significantly smaller compared to both the Medpor® (61.4 (59.0–84.0)%; *p*=0.049) and the Medpor® + PCL groups (69.7 (62.9–85.1)%; *p*=0.007). However, there was no statistically significant difference between the Medpor® group and the Medpor® + PCL group (*p*=0.2367) on day 4. The median (IQR) percentage of original wound size at day 20 was 6.0 (1.1–9.4)%, 11.6 (0–18.9)%, and 0 (0-0)% in the Medpor®, Medpor® + PCL, and Medpor® + PCL + PM groups, respectively.

### 3.2. Histological Staining

**Figure 4:**
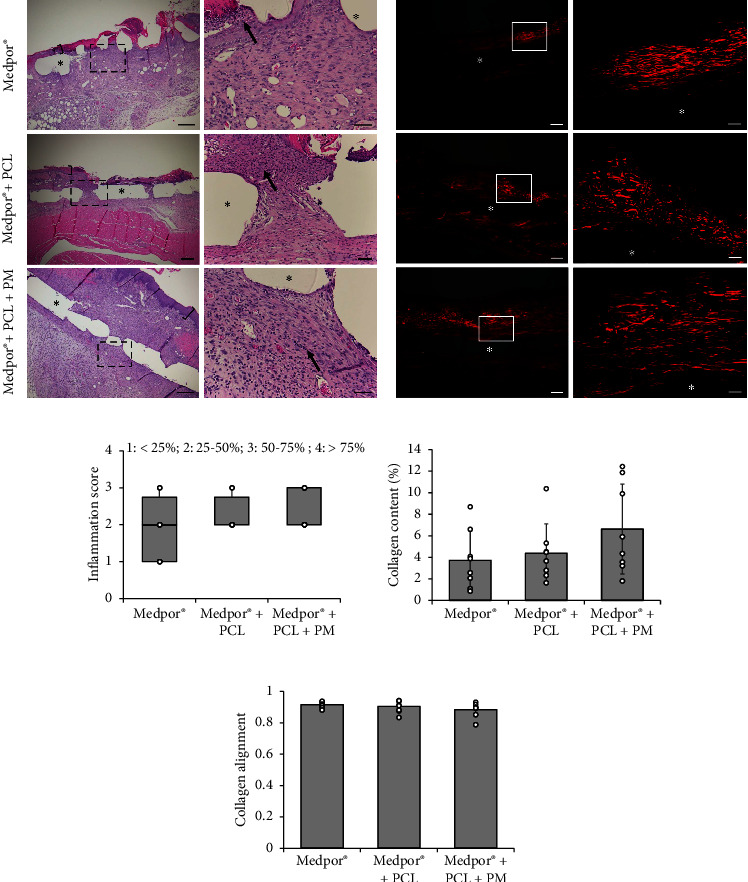
Representative images of stained wound tissue sections in C57BL/6 mice receiving Medpor®, Medpor® + PCL, and Medpor® + PCL + PM implants at day 21 postwounding. (a) H&E staining. Brackets indicate newly formed epithelium on top of the implant and arrows indicate inflammatory cells. Scale bars in left and right panels correspond to 250 and 50 *μ*m, respectively. (b) Picrosirius red staining under polarized light highlighting collagen fibers of the wound bed. Scale bars in left and right panels correspond to 200 and 50 *μ*m, respectively. Boxes in left panels denote zoomed regions displayed in right panels and void areas with asterisks denote implant location. (c) Inflammation score at day 21 postwounding in C57BL/6 mice receiving Medpor®, Medpor® + PCL, and Medpor® + PCL + PM implants. Boxes represent median and IQR. Mann–Whitney *U* tests were used in statistical analysis. (d) Collagen content percentage and (e) collagen fiber alignment at day 21 postwounding in C57BL/6 mice receiving Medpor®, Medpor® + PCL, and Medpor® + PCL + PM implants. Bars correspond to mean ± SD of individual data points. Tukey's HSD post hoc test was used in statistical analysis for *n* = 8 (number of wounds).

H&E staining was used to evaluate dermal remodeling and inflammatory response by determining the percentage of inflammatory cells (neutrophils, macrophages, and mast cells) in the wound area. Representative photos of histological sections of stained wounds taken 21 days postimplantation are shown in Figure 4(a). Inflammation was observed in all groups (arrows in Figure 4(a)), and the percentage of inflammatory cells in the wounds was quantified (Figure 4(c)). No significant difference in the inflammation score was observed between the groups (Medpor® *vs.* Medpor® + PCL: *p*=0.32; Medpor® *vs.* Medpor® + PCL + PM: *p*=0.21; Medpor® + PCL *vs.* Medpor® + PCL + PM; *p*=0.65). H&E staining also showed the formation of the newly formed epithelial layer over the implants (brackets in Figure 4(a)). In Medpor® + PCL + PM implants, the formed epithelium appears to offer more complete wound closure and a stratified epithelium, compared to that formed on Medpor® and Medpor® + PCL implants, which appear to offer sparse coverage and incomplete epithelialization. While H&E staining provided good indications of general tissue morphology, additional stains were employed to specifically examine collagen deposition, and the presence of an epidermal layer, blood vessels, and proliferating cells within and surrounding the implants.

Wound sections were subjected to Picrosirius red staining to examine the presence of collagen fibers and their organization under polarized light (Figure 4(b)). Fibers were detected and appeared red against a black background indicating collagen type I fibers, which primarily oriented parallel to the wound bed surface. The intensity of the deposited collagen, used to quantify the percentage of the wound areas covered with collagen for each group, did not show any significant differences (Medpor® *vs.* Medpor® + PCL: *p* = 0.92; Medpor® *vs.* Medpor® + PCL + PM: *p* = 0.21; Medpor® + PCL *vs.* Medpor® + PCL + PM; *p* = 0.38) among the wounds receiving different implants (Figure 4(d)). Similarly, the quantitative analysis of collagen alignment (Figure 4(e)) showed that the majority of collagen fibers in each of the three groups organized parallel to the wound bed orientation and that there was no statistical difference between the groups at 21 days postwounding (Medpor® *vs.* Medpor® + PCL: *p* = 0.47; Medpor® *vs.* Medpor® + PCL + PM: *p*=0.09; Medpor® + PCL *vs.* Medpor® + PCL + PM; *p*=0.35).

### 3.3. Immunofluorescence Staining

Wound tissue collected at 21 days postimplantation was evaluated using immunofluorescence staining: CD31 and *α*-SMA, E-CAD and cytokeratin, and Ki67 to assess tissue angiogenesis, epidermal closure, and cell proliferation, respectively.

**Figure 5:**
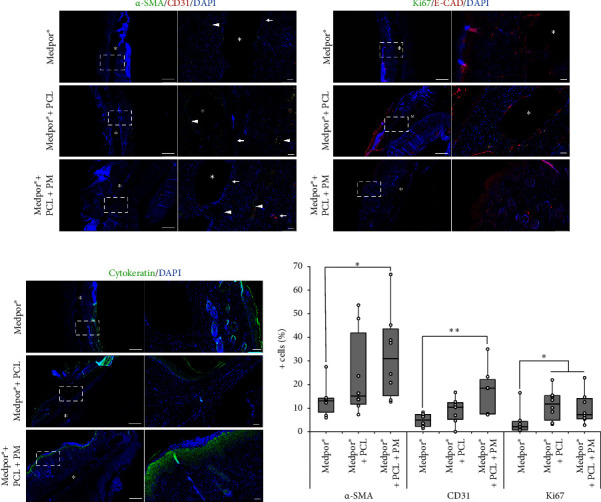
Representative images of immunofluorescent staining at day 21 postwounding for (a) *α*-SMA (green), CD31 (red), and DAPI (blue); (b) Ki67 (green), E-CAD (red), and DAPI (blue); and (c) cytokeratin (green) and DAPI (blue) of wound tissue sections in C57BL/6 mice receiving Medpor®, Medpor® + PCL, and Medpor® + PCL + PM implants. White boxes in left panels denote zoomed regions displayed in right panels. Void areas with asterisks denote implant location. Arrows indicate capillary networks and arrow heads indicate dermal fibroblasts. Long and short scale bars correspond to 500 and 50 *μ*m, respectively. (d) Quantitative analyses of positive *α*-SMA, CD31, and Ki67 cells at day 21 postwounding as a percentage of total cells in C57BL/6 mice receiving Medpor®, Medpor® + PCL, and Medpor® + PCL + PM implants. Boxes represent median and IQR. Mann–Whitney *U* tests were used in statistical analysis for *n* = 8 (number of wounds), ^∗^*p* < 0.05 and ^∗∗^*p* < 0.01.

The staining for CD31 and *α*-SMA revealed the presence of capillary networks (arrows in Figure 5(a)) and dermal fibroblasts (arrow heads in Figure 5(a)), respectively. Smooth muscle density showed an increasing trend with the subsequent addition of PCL and PM, with values of 13.1 (8.3–14.3)%, 15.1 (11.7–41.9)% and 31.1 (15.3–43.6)% for Medpor®, Medpor® + PCL, and Medpor® + PCL + PM groups, respectively (Figure 5(d)). However, this increase was only statistically significant between the Medpor® *vs.* Medpor® + PCL + PM (*p*=0.02) and not among Medpor® *vs.* Medpor® + PCL (*p*=0.23) and Medpor® + PCL *vs.* Medpor® + PCL + PM (*p*=0.38). A similar trend was observed for the number of newly formed capillaries, determined from CD31 staining, with the addition of PCL and then PM. Capillary density was particularly significantly higher in the Medpor® + PCL + PM group (18.5 (7.5–22.1)%) than in the Medpor® group (5.0 (2.3–7.4)%; *p*=0.005), indicating that the presence of both PCL and PM allowed better neovascularization.

Cell proliferation was assessed by staining with Ki67 antibody. Green fluorescence indicative of Ki67-positive cells was observed in Figure 5(b). The percentage of Ki67-positive nuclei per total cells was found to be 2.2 (0.7–4.4)%, 11.8 (4.9–15.3)%, and 7.3 (5.5–14.1)% for Medpor®, Medpor® + PCL, and Medpor® + PCL + PM groups, respectively (Figure 5(d)). The Medpor® group was significantly lower than the other two implant groups (Medpor® *vs.* Medpor® + PCL: *p*=0.02; Medpor® *vs.* Medpor® + PCL + PM: *p*=0.01), whilst there was no significant difference between them (Medpor® + PCL *vs.* Medpor® + PCL + PM; *p*=0.51). This suggests that the addition of PCL with or without the PM facilitates the proliferation of cells, a phase associated with previously mentioned tissue formation, re-epithelialization, and neovascularization.

Cytokeratin and E-CAD staining were performed to evaluate the effect of PCL and PM on keratinocytes and epithelial integrity. The data showed the expression of both markers across the wound gap over the implants in all three implant groups, as indicated by red fluorescence in Figure 5(b) and green fluorescence in Figure 5(c). Based on qualitative observation, Medpor® + PCL + PM had the highest expression of E-CAD and cytokeratin and the thickest layer of new epithelium.

## 4. Discussion

To advance biological implant development, it is crucial to establish an effective *in vivo* model for testing scaffolds derived from laboratory-based wound healing investigations. The evaluation of implants using an animal model relies heavily on its ability to replicate the intricate processes that occur during implant integration and wound healing in humans. The splinted excisional wound model in mice has proven valuable in mimicking human skin as it mitigates wound contraction, facilitating wound healing through epithelialization, granulation tissue formation, and angiogenesis [24, 29–33]. However, this model does not permit the study of implant efficacy in these animals. Thus, an objective of this study was to devise a splinted wound model capable of assessing the impact of implants on wound healing and to employ this model to evaluate the integration of previously developed implants within host tissues. To achieve this goal, we devised specific suturing techniques, wound sizes, implant sizes, splint sizes, and wound covers to optimize the incorporation of implants in the splinted wound model. Customized Medpor® implants were utilized, integrating an electrospun fiber layer with or without a protein mixture, and were placed within the developed model. Throughout the entire duration of the study, the implants exhibited successful incorporation in the animals, with no surgical complications. All implants facilitated wound size reduction, vascularization, epithelialization, and cell proliferation; however, Medpor®+ PCL + PM implants demonstrated faster wound closure, complete wound closure, and enhanced epithelialization, suggesting their potential for superior wound healing outcomes.

In this study, similar to our previous implant designs, we utilized electrospun PCL on Medpor® implants to introduce intricate fibrous structures resembling the native ECM [27]. Subsequently, a PM comprising collagen IV and laminin was adsorbed onto the fibers to enhance crucial processes for wound closure in this *in vivo* model, including cell attachment, migration, and proliferation [34]. These implants were incorporated in the splinted mouse model through meticulous suturing to ensure secure attachment to the surrounding skin while at the same time not damaging the implant, following the detailed steps outlined in the methods section. Previous studies have established the feasibility of testing the efficacy of various implant-free treatments on wound healing in mice using similar excisional wound models. For instance, studies by Li et al. and Bruna et al. demonstrated that mesenchymal cells enhance the wound microenvironment [35, 36]. Similarly, studies by Wang et al., Dong et al., and Gordts et al. highlighted the beneficial effects of telocytes, recombinant proteins, and high-density lipoproteins, respectively, on wound healing [37–39]. Conversely, and in similar studies, investigations by Park et al. and Nauta et al. concluded that platelet-derived growth factor-BB and mast cells, respectively, do not accelerate healing in mouse excisional wounds [28, 40]. Another study by Chen et al. focused on evaluating the feasibility of hydrogel dressings using a similar model for wound healing [41]. However, our study diverges from the aforementioned research by specifically addressing the incorporation of structural implants and their wound healing capacity within the splinted wound model. Moreover, our methods effectively prevent the exposure or detachment of the implants and splints, as well as the removal of bandages by the mice.

The process of dermal wound healing in rodents, like in humans, is a dynamic and interconnected series of events. Inflammation is triggered immediately after an injury, and inflammatory cells release various factors that promote cell proliferation, angiogenesis, and migration [35, 37, 42]. Subsequently, during the remodeling phase, re-epithelialization and the formation of new tissue take place, involving both apoptosis and the reorganization of matrix proteins, such as collagen [24]. In our study, histological examination using H&E staining provided valuable insight into overall tissue morphology and indicated the presence of inflammatory cells within and surrounding the implanted scaffolds (Figures 4(a) and 4(c)). As expected, the foreign body response was observed, as the immune system recognized the Medpor® implants as an inert foreign object and recruited inflammatory cells accordingly [43]. This finding aligns with previous reports by Paxton et al. who observed the formation of foreign body giant cells as recruited mast cells and macrophages failed to digest the macroscopic polymer structures of the Medpor® when it was subcutaneously implanted in rats for 8 weeks [44]. In addition, collagen deposition around the implants was observed in our study as part of the reparative phase, indicating the regulation of the local tissue reaction and the control of fibroblast activity triggered by the inflammatory response [36, 43–45]. However, similar collagen content percentages and orientation (Figures 4(b), 4(d), and 4(e)) suggested comparable remodeling intensities between the different implants 21 days postimplantation.

To ensure the inclusion of advanced stages of healing, we chose a study duration of 21 days based on previous findings indicating that 5 mm wounds in 7–8-week-old C57BL/6 mice typically heal within this time frame [46]. Moreover, the differentiation of the newly formed epidermis, as reported by Wiksman et al. was observed to occur within 18 days [47]. Over the course of the 21-day period, it became evident, through visual examination of wound images, that the three implant groups underwent typical wound healing processes and progressed through different phases of healing. However, wounds receiving both PCL and PM incorporated onto the Medpor® demonstrated faster closure rates and achieved more complete closure compared to wounds receiving Medpor® alone and Medpor® + PCL implants, as depicted in Figures 2, 3, 4, and 5. The Medpor® and Medpor® + PCL groups displayed larger, less contracted wounds, and the scab formed on the wound areas appeared to be more pronounced and persisted for a longer duration (up to 20 days, Figure 2). This is consistent with the work of Li et al. where wounds left untreated displayed similar slower healing trends compared to wounds treated with mesenchymal stem cells [35]. H&E staining, analogously, showed greater wound re-epithelialization and the presence of normal epithelial layers exclusively in the Medpor® + PCL + PM implant group (Figure 4(a)). This observation was further confirmed by E-CAD and cytokeratin staining, which exhibited the highest expression and indicated the thickest epithelium in the Medpor® + PCL + PM implant group (Figure 5). Such results were expected, considering that the addition of proteins makes implant surfaces favorable for the attachment and migration of epithelial cells [27, 38, 39]. The formation of blood vessels is an indispensable stage of wound repair, as it is essential to restore oxygen via blood supply to damaged tissues [38, 48]. Previous *in vivo* models have established that CD31 and *α*-SMA expressions are the characteristic markers of this process [38]. Our study demonstrated increasing trends of CD31 and *α*-SMA expressions with the sequential addition of electrospun PCL followed by the inclusion of the PM, suggesting a positive impact on angiogenesis through their incorporation with Medpor® implants (Figure 5(d)).

Since the primary objective of this research entailed assessing the impacts of implants composed solely of Medpor® (a commonly used implant material in clinical practice) versus those incorporating PCL and PCL + PM, findings from an untreated animal cohort were not included herein. This may be a study limitation as alterations noted in wound healing processes may not be exclusively attributable to variations in the implants. Statistical analysis did not reveal any significant differences among the implants in terms of various evaluated parameters. However, the rate of wound closure emerged as the most differentiating factor and is considered the primary outcome in excisional wound studies [46]. Another potential limitation of our study was the inability to comprehensively assess angiogenesis, cell proliferation, inflammatory response, and matrix deposition at multiple time points throughout the study duration. As a result, there were no significant differences necessarily observed in the performance of these implants for these specific processes at 21 days postimplantation. Previous *in vivo* excisional wound studies conducted by Bruna et al., Orlowski et al., and Short et al. reported disparities in collagen content, blood vessel count, and inflammatory cell numbers among their different study groups at 12 days, 6 days, and 7 days postwounding, respectively [36, 49, 50]. These differences were all detected within a significantly shorter duration than the 21-day time frame. However, we deliberately avoided employing invasive protocols such as wound biopsies or increasing the number of animals for multiple time point assessments. This decision was based on our study's objective, which did not involve an in-depth examination of the distinct phases of wound healing. Instead, our study aimed to provide an overview of the healing of 5 mm wounds treated with various implants in a simplified manner. This approach establishes a foundation for future studies to explore additional factors within the complex landscape of healing methods.

In summary, our analysis of wound images revealed that wounds treated with Medpor® + PCL + PM implants exhibited the fastest healing, with complete wound closure achieved between days 12 and 14. Histological examination using H&E staining and epithelium-related staining confirmed the complete wound closure with these implants at the end of the 21-day period (Table 1). Moreover, the sequential addition of PCL and PM was found to be associated with the formation of blood vessels, as demonstrated by analogous findings from angiogenic-related staining. However, the addition of PCL and PM did not significantly enhance parameters related to collagen deposition and inflammatory response.

## 5. Conclusion

This novel study provides compelling evidence that the addition of both PCL and PM to Medpor® implants offers important advantages in wound healing. These implants demonstrated accelerated wound closure rates, improved epithelialization, and positive effects on angiogenesis, highlighting their potential as valuable components in the field of wound healing applications. By introducing a splinted wound model that integrates implants, our study contributes to the development of an effective preclinical testing framework for implant scaffolds. This foundation will pave the way for future investigations to explore additional components, ultimately advancing our knowledge of implant efficacy and facilitating the development of enhanced strategies for wound healing.

## Figures and Tables

**Table 1:** Summary of studied wound healing mechanisms and their corresponding outcome.

Wound healing mechanism	Outcome
Wound closure	PM and PCL addition to Medpor® accelerates closure rates
Inflammation	No significant differences observed between groups 21 days postwounding
Collagen deposition	No significant differences observed between groups 21 days postwounding
Angiogenesis	Sequential addition of PCL then PM to Medpor® enhances angiogenesis
Epithelialization	PM and PCL addition to Medpor® promotes more complete closure and a thicker, stratified epithelium
Proliferation	PCL addition with or without PM to Medpor® facilitates proliferation 21 days postwounding

## Data Availability

The data used to support the findings of this study are available from the corresponding author upon request.

## References

[1] Velnar T., Bailey T., Smrkolj V. (2009). The wound healing process: an overview of the cellular and molecular mechanisms. *Journal of International Medical Research*.

[2] Koschwanez H. E., Broadbent E. (2011). The use of wound healing assessment methods in psychological studies: a review and recommendations. *British Journal of Health Psychology*.

[3] Aragona M., Dekoninck S., Rulands S. (2017). Defining stem cell dynamics and migration during wound healing in mouse skin epidermis. *Nature Communications*.

[4] Díaz-García D., Filipová A., Garza-Veloz I., Martinez-Fierro M. L. (2021). A beginner’s introduction to skin stem cells and wound healing. *International Journal of Molecular Sciences*.

[5] Pang C., Ibrahim A., Bulstrode N. W., Ferretti P. (2017). An overview of the therapeutic potential of regenerative medicine in cutaneous wound healing. *International Wound Journal*.

[6] Romanelli M., Miteva M., Romanelli P., Barbanera S., Dini V. (2013). Use of diagnostics in wound management. *Current Opinion in Supportive and Palliative Care*.

[7] Yunusov K. E., Sarymsakov A. A., Jalilov J. Z. O., Аtakhanov A. A. O. (2021). Physicochemical properties and antimicrobial activity of nanocomposite films based on carboxymethylcellulose and silver nanoparticles. *Polymers for Advanced Technologies*.

[8] Li P., Ruan L., Jiang G. (2022). Design of 3D polycaprolactone/*ε*-polylysine-modified chitosan fibrous scaffolds with incorporation of bioactive factors for accelerating wound healing. *Acta Biomaterialia*.

[9] Kolimi P., Narala S., Nyavanandi D., Youssef A. A. A., Dudhipala N. (2022). Innovative treatment strategies to accelerate wound healing: trajectory and recent advancements. *Cells*.

[10] Wu J., Mao Z., Tan H., Han L., Ren T., Gao C. (2012). Gradient biomaterials and their influences on cell migration. *Interface focus*.

[11] Storrie H., Mooney D. J. (2006). Sustained delivery of plasmid DNA from polymeric scaffolds for tissue engineering. *Advanced Drug Delivery Reviews*.

[12] Wellisz T. (1993). Clinical experience with the Medpor porous polyethylene implant. *Aesthetic Plastic Surgery*.

[13] Hashem F. K., Homsi M. A., Mahasin Z. Z., Gammas M. A. (2001). Laryngotracheoplasty using the Medpor implant: an animal model. *Journal of Otolaryngology*.

[14] Lee D. J., Kwon J., Kim Y.-I., Kwon Y. H., Min S., Shin H. W. (2020). Coating Medpor® implant with tissue-engineered elastic cartilage. *Journal of Functional Biomaterials*.

[15] Laschke M., Augustin V., Kleer S., Tschernig T., Menger M. (2014). Locally applied macrophage-activating lipopeptide-2 (MALP-2) promotes early vascularization of implanted porous polyethylene (Medpor®). *Acta Biomaterialia*.

[16] Arrieta M. P., Leonés Gil A., Yusef M., Kenny J. M., Peponi L. (2020). Electrospinning of PCL-based blends: processing optimization for their scalable production. *Materials*.

[17] Malikmammadov E., Tanir T. E., Kiziltay A., Hasirci V., Hasirci N. (2018). PCL and PCL-based materials in biomedical applications. *Journal of Biomaterials science*.

[18] Dash T. K., Konkimalla V. B. (2012). Polymeric modification and its implication in drug delivery: poly-*ε*-caprolactone (PCL) as a model polymer. *Molecular Pharmaceutics*.

[19] Mujica-Garcia A., Navarro-Baena I., Kenny J. M., Peponi L. (2014). Influence of the processing parameters on the electrospinning of biopolymeric fibers. *Journal of Renewable Materials*.

[20] Lv H., Tang D., Sun Z. (2020). Electrospun PCL-based polyurethane/HA microfibers as drug carrier of dexamethasone with enhanced biodegradability and shape memory performances. *Colloid and Polymer Science*.

[21] Iorio V., Troughton L. D., Hamill K. J. (2015). Laminins: roles and utility in wound repair. *Advances in Wound Care*.

[22] Dong C., Lv Y. (2016). Application of collagen scaffold in tissue engineering: recent advances and new perspectives. *Polymers*.

[23] Breitkreutz D., Koxholt I., Thiemann K., Nischt R. (2013). Skin basement membrane: the foundation of epidermal integrity—BM functions and diverse roles of bridging molecules nidogen and perlecan. *BioMed Research International*.

[24] Dunn L., Prosser H. C., Tan J. T., Vanags L. Z., Ng M. K., Bursill C. A. (2013). Murine model of wound healing. *Journal of Visualized Experiments*.

[25] Wong V. W., Sorkin M., Glotzbach J. P., Longaker M. T., Gurtner G. C. (2011). Surgical approaches to create murine models of human wound healing. *BioMed Research International*.

[26] Gadalla D., Goldstein A. S. (2020). Improving the osteogenicity of PCL fiber substrates by surface-immobilization of bone morphogenic protein-2. *Annals of Biomedical Engineering*.

[27] Gadalla D., Tchoukalova Y. D., Lott D. G. (2022). Regenerating airway epithelium using fibrous biomimetic basement membranes. *Journal of Biomedical Materials Research Part A*.

[28] Park S. A., Raghunathan V. K., Shah N. M. (2014). PDGF-BB does not accelerate healing in diabetic mice with splinted skin wounds. *PLoS One*.

[29] Galiano R. D., Michaels V., Dobryansky J. M., Levine J. P., Gurtner G. C. (2004). Quantitative and reproducible murine model of excisional wound healing. *Wound Repair and Regeneration*.

[30] Jimi S., De Francesco F., Ferraro G. A., Riccio M., Hara S. (2017). A novel skin splint for accurately mapping dermal remodeling and epithelialization during wound healing. *Journal of Cellular Physiology*.

[31] Huang J., Wu J., Wang J. (2023). Rock climbing-inspired electrohydrodynamic cryoprinting of micropatterned porous fiber scaffolds with improved msc therapy for wound healing. *Advanced Fiber Materials*.

[32] Huang J., Yang R., Jiao J. (2023). A click chemistry-mediated all-peptide cell printing hydrogel platform for diabetic wound healing. *Nature Communications*.

[33] Luo P., Huang R., Wu Y. (2023). Tailoring the multiscale mechanics of tunable decellularized extracellular matrix (dECM) for wound healing through immunomodulation. *Bioactive Materials*.

[34] Park S., Gonzalez D. G., Guirao B. (2017). Tissue-scale coordination of cellular behaviour promotes epidermal wound repair in live mice. *Nature Cell Biology*.

[35] Li H., Ziemer M., Stojanovic I. (2022). Mesenchymal stem cells from mouse hair follicles reduce hypertrophic scarring in a murine wound healing model. *Stem Cell Reviews and Reports*.

[36] Bruna F., Contador D., Conget P., Erranz B., Sossa C. L., Arango-Rodríguez M. L. (2016). Regenerative potential of mesenchymal stromal cells: age-related changes. *Stem Cells International*.

[37] Wang L., Song D., Wei C. (2020). Telocytes inhibited inflammatory factor expression and enhanced cell migration in LPS-induced skin wound healing models in vitro and in vivo. *Journal of Translational Medicine*.

[38] Dong Y., Zhu W., Lei X. (2022). Treatment of acute wounds with recombinant human-like collagen and recombinant human-like fibronectin in C57bl/6 mice individually or in combination. *Frontiers in Bioengineering and Biotechnology*.

[39] Gordts S. C., Muthuramu I., Amin R., Jacobs F., Geest B. D. (2014). The impact of lipoproteins on wound healing: topical HDL therapy corrects delayed wound healing in apolipoprotein E deficient mice. *Pharmaceuticals*.

[40] Nauta A. C., Grova M., Montoro D. T. (2013). Evidence that mast cells are not required for healing of splinted cutaneous excisional wounds in mice. *PLoS One*.

[41] Chen K., Sivaraj D., Davitt M. F. (2022). Pullulan‐collagen hydrogel wound dressing promotes dermal remodelling and wound healing compared to commercially available collagen dressings. *Wound Repair and Regeneration*.

[42] Masson‐Meyers D. S., Andrade T. A., Caetano G. F. (2020). Experimental models and methods for cutaneous wound healing assessment. *International Journal of Experimental Pathology*.

[43] Ibrahim M., Bond J., Medina M. A. (2017). Characterization of the foreign body response to common surgical biomaterials in a murine model. *European Journal of Plastic Surgery*.

[44] Paxton N. C., Dinoro J., Ren J. (2021). Additive manufacturing enables personalised porous high-density polyethylene surgical implant manufacturing with improved tissue and vascular ingrowth. *Applied Materials Today*.

[45] Birch M., Tomlinson A., Ferguson M. W. (2005). Animal models for adult dermal wound healing. *Fibrosis Research: Methods and Protocols*.

[46] Wang X., Ge J., Tredget E. E., Wu Y. (2013). The mouse excisional wound splinting model, including applications for stem cell transplantation. *Nature Protocols*.

[47] Braiman-Wiksman L., Solomonik I., Spira R., Tennenbaum T. (2007). Novel insights into wound healing sequence of events. *Toxicologic Pathology*.

[48] Demidova-Rice T. N., Durham J. T., Herman I. M. (2012). Wound healing angiogenesis: innovations and challenges in acute and chronic wound healing. *Advances in Wound Care*.

[49] Orlowski P., Zmigrodzka M., Tomaszewska E. (2018). Tannic acid-modified silver nanoparticles for wound healing: the importance of size. *International Journal of Nanomedicine*.

[50] Short W. D., Rae M., Lu T. (2023). Endogenous interleukin-10 contributes to wound healing and regulates tissue repair. *Journal of Surgical Research*.

